# Management of heterotopic ossification and restricted forearm rotation after tension band wiring for olecranon fracture

**DOI:** 10.1007/s11751-014-0197-8

**Published:** 2014-07-26

**Authors:** Karuppaiah Karthik, Ramon Tahmassebi, Adel Tavakkolizadeh, Jonathan Compson

**Affiliations:** Upper Limb Unit, Department of Orthopaedic Surgery, King’s College Hospital, Denmark Hill, London, SE59RS UK

**Keywords:** Olecranon fracture, Tension band wiring, Heterotopic ossification, Restricted forearm rotation, Proximal radio-ulnar synostosis

## Abstract

A 32-year-old lady presented to our clinic with persistent painful restriction of her dominant forearm movements for three months after tension band wiring of olecranon. She had full elbow flexion and extension; however, her forearm rotations were restricted and painful. Investigations revealed prominent tips of the wire, eroding the radial tuberosity with heterotopic ossification between the radius and ulna. As there was no synostosis, the patient had implant exit. During surgery, before implant removal, examination under anaesthesia revealed a mechanical block of the rotation beyond 30° on pronation and supination from neutral. However, after the removal of implant, the mechanical block eased off and with gentle manipulation, full pronation and supination were achieved. At the final follow-up at 6 months, the patient had full pain-free forearm rotation with regression of heterotopic ossification. Our case report highlights the importance of intra-operative assessment of wire tips at full supination and pronation, and in patients with restricted forearm rotation, CT scan may be needed to assess the position of the hardware is essential as it can progress to synostosis. In cases with prominent hardware, removal of the implant may suffice if performed before the development of synostosis

## Introduction

Tension band wiring (TBW) is the gold standard technique for treatment of simple intra-articular fractures of the olecranon [1, 2]. A recent study evaluated various configurations of wire placement and suggested transcortical wires penetrating the anterior cortex of the ulna rather than intramedullary wires to reduce complications related to wire migration [2]. Most complications reported in the literature are related to wire migration and prominence. However, non-union, infection, post-traumatic arthrosis, reflex sympathetic dystrophy and ulnar nerve palsy have all been described [2–4]. Reported cases of restricted forearm rotation [5, 6] and proximal radio-ulnar synostosis [7, 8] following TBW are less common, and the cases described offer little detail regarding the identification and management of these rare complications. We describe the management of a patient who presented with painful restriction of forearm movements and progressive proximal radio-ulnar synostosis due to heterotopic ossification following TBW of an olecranon fracture.

## Case report

**Fig. 1:**
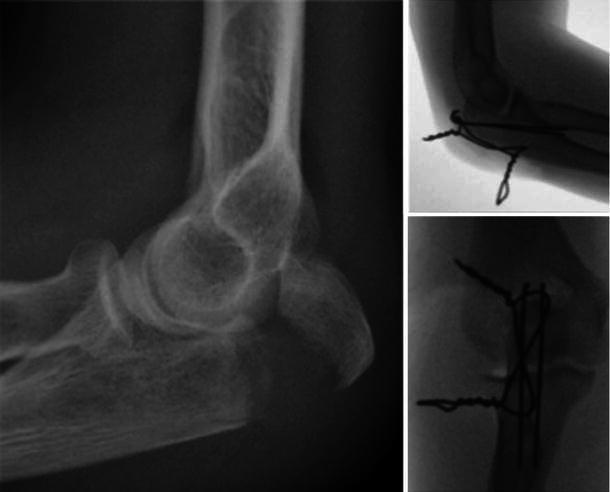
Radiograph showing simple transverse olecranon fracture (*left*), intra-operative image intensifier view showing good fracture reduction

**Fig. 2:**
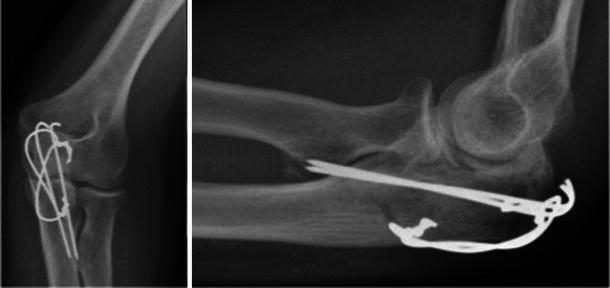
Radiographs at 2-month follow-up showed heterotopic ossification around the tips of the wires

A 32-year-old lady was referred to our clinic with a diagnosis of myositis ossificans and restricted forearm movements following TBW of her olecranon. Three months earlier, the patient had sustained an isolated, closed, transverse olecranon fracture of her dominant (left) side (Fig. 1). Her post-operative recovery was initially uneventful, but she failed to regain function due to persistent elbow pain and stiffness. She therefore underwent a course of intensive physiotherapy. Two months post-surgery, her range of movement was between 30^°^ and 90^°^ in flexion/extension and 10^0^ each of pronation and supination when measured from a mid-prone position. Movements throughout the range of movement were accompanied by a painful, audible and palpable clicking arising from the elbow joint. The pain was severe during the early phases of rehabilitation and subsequently improved, but did not completely resolve. Check X-ray at 2-month follow-up showed heterotopic ossification around the tips of the wires (Fig. 2) and indomethacin 75 mg once a day was started.

**Fig. 3:**
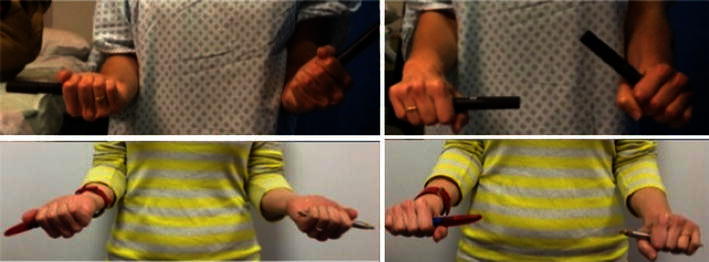
Clinical picture demonstrating 30^°^ of pronation and supination (preoperative, *top*), and normal supination and pronation at the final follow-up at 6 months

At 3 months after surgery, the patient presented to our clinic. She had regained a full range of movement in flexion and extension (0^°^–140^°^). However, she still had a significant restriction of rotatory forearm movements (30^°^ supination and 30^°^ pronation), which were associated with pain and crepitation (Fig. 3). She complained of severe difficulty in using her hand for all activities of daily living. The surgical wound and fracture had both healed satisfactorily. An X-ray taken at 3 months after surgery showed the tips of the wires near radial tuberosity with surrounding heterotopic ossification. A CT scan of the elbow was organised to investigate the possibility of a mechanical block to rotatory movements and to evaluate the scale of heterotopic ossification.

**Fig. 4:**
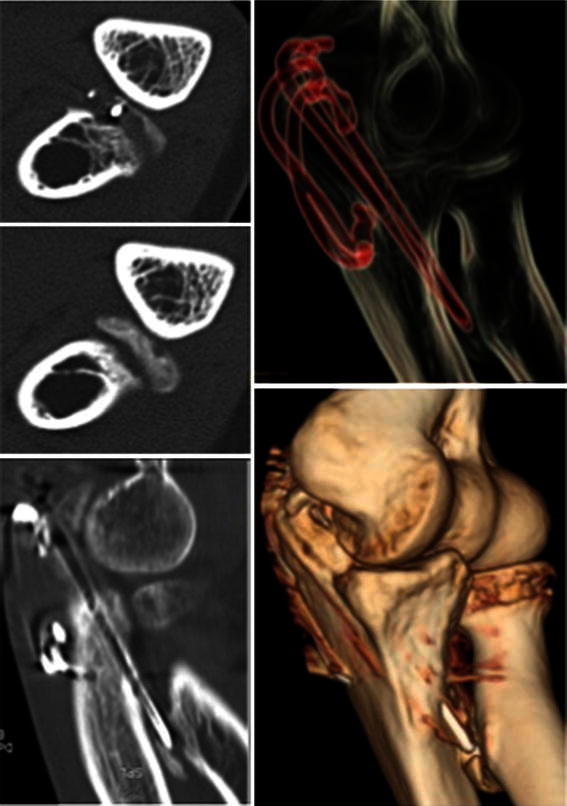
CT scan confirmed that the prominent ends of the wires were eroding the radial tuberosity with secondary heterotopic ossification between the radius and ulna. In the coronal view (*left*, *middle*). there was 1-mm gap between the heterotopic ossification and proximal ulnar shaft

**Fig. 5:**
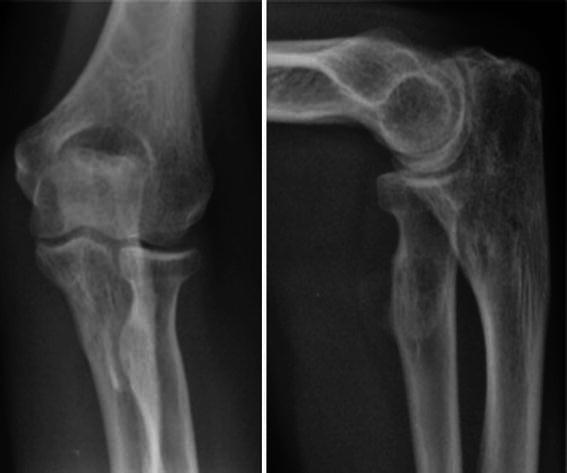
Radiographs at final follow-up showed good fracture healing and regression of heterotopic ossification

The scan confirmed that the prominent ends of the wires were eroding the radial tuberosity with secondary heterotopic ossification between the radius and ulna (Fig. 4). As the synostosis was not yet complete, the patient was listed for removal of metalwork. At surgery, examination under anaesthesia revealed a mechanical block of the rotation beyond 30^0^ on pronation and supination. However, after the removal of implants, the block was easily overcome with gentle manipulation. Full passive pronation and supination were restored intra-operatively. At the final follow-up at 6 months, the patient had full, pain-free forearm rotation (Fig. 3). The radiographs at final follow-up showed regression of heterotopic ossification (Fig. 5).

## Discussion

Tension band wiring of the olecranon is a commonly performed technique and can be performed safely if careful attention is paid during the insertion of wires [5, 6]. The transcortical technique, though associated with fewer complications such as wire migration, soft tissue irritation and reduced rate of re-operation [2], can lead to major complications if the tips of the wires are left prominent beyond the anterior cortex of the ulna [5–9]. A cadaveric study showed that even 2–3 mm of protrusion caused impingement over the biceps tendon and supinator muscle during pronation [5].

Restriction of forearm rotation can occur after surgery if the wire or screw is directed radially and is left too prominent beyond the far cortex. In the case we report, the wire may have penetrated the proximal radial cortex, leading to severe stiffness in the first 2 months. During physiotherapy, the wire may have disengaged allowing an improved ROM but simultaneously eroding the adjacent radial cortex (Fig. 4).

There are two reports in the literature evaluating causes and preventative measures for this rare complication [5, 6]. Candal-Couto et al. [5] reported 2 cases, one with impaired forearm rotation following soft tissue irritation and the other with no forearm rotation as the tip of the most radial wire had penetrated the radial neck. The report did not offer further clinical or radiological details. The authors then performed a cadaveric study to assess the safety of the transcortical technique. They suggested that limitation of forearm rotation was more likely to occur if the forearm was pronated during the wire insertion, which may have been the case in our patient. Review of the intra-operative images showed that the wires were inserted with the arm in pronation. They also suggested that the entry point of the wires should have been more radial and directed towards the ulnar shaft. A study by Matthews et al. [6] investigated this phenomenon further using a computerised simulation model with three-dimensional computed tomography reconstruction of the elbow. The conclusions from their study were similar to the cadaveric study of Candal-Couto et al. [5]. Both these reports stressed the importance of clinical and radiological assessment of forearm rotation irrespective of method of olecranon fixation. Besides, the authors stress the point that the direction of wires should be proximal lateral to distal medial.

Proximal radio-ulnar synostosis following TBW has been described in the literature in three patients. In one report with two patients, clinical and radiological data were very limited [7]. In the second report, the patient presented 1 year after surgery with a complete block of pronation and supination [8]. The tips of the wires were resting on the radial cortex, and the images showed erosion of the part of radial cortex with heterotopic ossification. Although X-rays taken in the early post-operative phase showed heterotopic calcification around the proximal radius, the patient developed synostosis at 13 months following surgery. The authors concluded that wires impinging on the radial cortex could cause direct periosteal stimulation leading to heterotopic ossification and consequent synostosis.

Fortunately, in our patient, we identified the problems early before the development of synostosis. The CT scan of our patient at 3 months showed a 1-mm gap between the ulna and the heterotopic bone arising from the radius (Fig. 4). This factor may have been important in the recovery of her range of movement through manipulation alone. Our report addresses the gap in the literature; in patients with heterotopic ossification without synostosis, removal of the wire followed by manipulation and physiotherapy can restore full function with regression of heterotopic bone formation.

## Conclusion

In patients undergoing TBW for olecranon fractures, it is essential to make a full intra-operative assessment of forearm rotation and to radiologically ensure the tips of the wires are not impinging in supination or pronation. The direction of wires from proximal radial to distal ulnar will reduce the chance of impingement. Restriction of forearm movements following surgery should be evaluated with CT scan. Removal of the offending hardware and gentle manipulation is enough if performed before the space between radius and ulna is obliterated.
